# Home blood-pressure measurement for the diagnosis and monitoring of arterial hypertension by French general practitioners: a cross-sectional survey in the Auvergne region

**DOI:** 10.1186/s12875-020-01358-9

**Published:** 2021-01-04

**Authors:** Mangiavillano Xavier, Tréfond Jéromine, Plaquevent-Hostache Guillaume, Tanguy Gilles, Bœuf-Gibot Sylvaine, Mulliez Aurélien, Vorilhon Philippe

**Affiliations:** 1 Department of General Medicine, UFR Medicine, Clermont Auvergne University, 28, place Henri Dunant, 63000 Clermont-Ferrand, France; 2 Université Clermont Auvergne, Unité de recherche ACCePPT, Clermont-Ferrand, France; 3 Cardio-Pneumology Medical Clinic, 63830 Durtol, France; 4 Clinical Research and Innovation Delegation, Clermont-Ferrand University Hospital, 58 Rue Montalembert, 63003 Clermont-Ferrand, France

**Keywords:** Arterial hypertension, Blood pressure, Home blood pressure measurement, Guidelines, General practitioners, Cross-sectional survey

## Abstract

**Background:**

Home blood-pressure measurement (HBPM) is recommended for the diagnosis of hypertension and monitoring of patients with hypertension. Since 2013, the French National Health Insurance Body (FNHIB) has promoted HBPM to general practitioners (GPs). The objective of the study was to evaluate the practice of HBPM by French GPs to diagnose and monitor hypertension, 3 years after the beginning of the FNHIB campaign.

**Methods:**

We conducted a postal survey from 7 March to 31 May 2016. All of the 1040 GPs practising in the Auvergne region in France were invited to participate, using a self-reporting questionnaire. We obtained information on the characteristics of the GPs, and their practice regarding the use of HBPM. Use of HBPM was reported as “never, occasionally, regularly and systematically”. Frequency of HBMP use was analysed using multivariate ordered logistic regression model.

**Results:**

A total of 569 (54.7%) GPs responded to the survey. They were 50.3 (± 11.5) years old, 241 (43.0%) were female, and 352 (62.7%) worked in urban areas. Among them, 530 (94.5%) reported the use of HBPM for diagnosis and 519 (92.5%) for monitoring hypertension. To diagnose hypertension, younger GPs (OR = 0.97; 95% CI: 0.95–0.98), GPs practising in a group (OR = 1.45; 95% CI: 1.00–2.10) and in an MHC (OR = 2.09; 95% CI: 1.15–3.78), as compared to GPs with individual practices, and Clinical Tutors, as compared to non-Clinical Tutors, (OR = 1.92; 95% CI: 1.33–2.79) reported more frequent use of HBPM. To manage hypertension, female GPs, as compared to male GPs, (OR=1.47; 95% CI: 1.04–2.06), younger GPs (OR = 0.98; 95% CI: 0.97–0.99), and Clinical Tutors (OR = 1.90; 95% CI: 1.31–2.75) reported more frequent use of HBPM.

**Conclusions:**

Our survey reveals that 3 years after the introduction of the FNHIB incentives, the use of HBPM by participating GPs to diagnose and manage hypertension is well established. A larger survey of GPs from other regions would make it possible to verify whether our results can be extrapolated to France as a whole.

**Supplementary Information:**

The online version contains supplementary material available at 10.1186/s12875-020-01358-9.

## Key messages

- 530 (94.5%) of 561 GPs reported using HBPM to diagnose hypertension.
- 519 (92.5%) reported its use for monitoring.
- Female GPs and Clinical Tutors were more likely to use HBPM
- 89.2% of the 501 GPs who lent out a device recommended the appropriate rules of use

## Background

Home blood-pressure measurement (HBPM) is recommended for the diagnosis of hypertension. The European Society of Hypertension (ESH) defines hypertension as a systolic blood pressure (SBP) at least 135 and/or diastolic blood pressure (DBP) at least 85 mmHg, taking an average over 3–6 days of blood pressure (BP) readings [1]. HBPM is also recommended to monitor hypertension in patients. BP control is defined as having a SBP < 140 mmHg and a DBP < 90 mmHg in all patients, which should be under 130/80 mmHg if tolerated. These targets are lower for patients under 65 years old or with diabetes (SBP < 130 mmHg). For older patients, DBP should be less than 80 mmHg [1]. HBPM, in conjunction with co-interventions as an adjunct to normal care, results in a significant additional reduction in BP after 12 months [2]. It improves the quality of the doctor–patient relationship and enables patients both to gain a better understanding of their condition and to take a more active role in its treatment [1, 3, 4]. Nevertheless, the use of HBPM remains low in developing countries [5, 6], and infrequent in developed countries including France [7–11]. To our best knowledge, there are no data available on the practice of HBPM for the Auvergne region. The barriers to the use of HBPM are numerous, but the cost of the device and non-reimbursement seem to be an important one [8, 12]. Since 2013, the French National Health Insurance Body (FNHIB) has taken measures to promote the use of HBPM among General practitioners (GPs). Along with the delivery of their summary recommendations is an automatic device (Microlife BP A200®), available for GPs to lend free of charge to their patients [13]. Recommendations on the management of hypertension have also been published by the French State Health Authority (HAS) and the French League Against Hypertension (CFLHTA) [14]. Our hypothesis is that such incentives have increased the use of HBPM for screening and monitoring patients with hypertension by French GPs in recent years.

The objective of our study was to evaluate the use of HBPM to diagnose and monitor patients with hypertension by GPs in the Auvergne region. We also calculated the percentage of GPs who ordered the HBPM device provided by the FNHIB, and the percentage of GPs who were able to use this device to initiate HBPM.

## Methods

### Study type

We conducted a cross-sectional survey, performed from 7 March to 31 May 2016 and involving GPs practising in the Auvergne region. Auvergne was used as a pilot region for the whole of France to assess the experience of the measures introduced by FNHIB in 2013.

### Population

The survey took place in the Auvergne region, which consists of 4 counties with a total population of 1,364,156. According to the National Medical Council, 1263 GPs were registered in the region during the period of the survey. To be eligible to participate, GPs were required to be practising as primary care physicians and to be responsible for the longitudinal care of the local and community-based population. GPs with particular specialisms (homeopathy, osteopathy, acupuncture, angiology, thermal spa medicine, occupational medicine, and specialised accident and emergency) were not included in this study, accounting for 261 GPs. Eleven GPs had retired or changed occupation. In total, 1040 GPs were contacted.

### Questionnaire

We developed a questionnaire survey designed on the basis of a literature review of international recommendations, epidemiological data, and surveys of national practices [1, 4, 14]. We first tested it on a panel of 20 GPs to improve content, understanding, and ease of use. The questionnaire comprised three parts:

- details of the GP, including age, sex, location and county of practice, type of practice, and status as a French university Clinical Tutor.
- evaluation of the GP’s practice regarding use of HBPM (frequency, method of BP control, ownership of an HBPM device, ownership of an Ambulatory Blood Pressure Measurement (ABPM) device, method of diagnosing hypertension, benefits of HBPM [masked hypertension, white-coat syndrome, treatment adaptation], method of monitoring patients with hypertension, frequency of use of HBPM to diagnose and monitor hypertension). Frequency was defined as “never, occasionally, regularly, and systematically”. This corresponds to an increasing frequency of use and was analysed as such.

### Study procedure

HBPM must be performed using a standardised measurement protocol that differs according to scientific societies. It is recommended to use automatic armband model devices with recording of measurements. In France, the French State Health Authority (HAS) and CFLHTA recommend 3 measurements in the morning at breakfast and 3 in the evening before bed for 3 consecutive days (the ‘rule of three’) [14]. Measurements should be spaced at 1-min intervals, after a few minutes of rest. This schedule has not been validated scientifically and differs from the guidelines of the European Society of Hypertension [15].

### Outcome measures

The primary outcome was the percentage of GPs who used HBPM to diagnose hypertension and to monitor patients with hypertension. The secondary outcomes were the percentage of GPs who ordered the HBPM device provided by the FNHIB, and the percentage of GPs who were enabled by this device to initiate the use of HBPM.

### Statistical analysis

The data were analysed using Stata V12 (StataCorp LLC, College Station, TX, USA). All statistical analyses were performed using two-tailed tests with a Type-I error of α = 5%. The proportion of GPs who used HBPM to diagnose and monitor hypertension was expressed as a percentage with an associated 95% confidence interval (CI); the proportion of GPs using an ABPM device was expressed similarly. Analyses of the use of HBPM (Never / Occasionally / Regularly / Systematically) to diagnose and monitor were performed using chi-squared test (or Fisher’s exact test where appropriate) and Analysis of variance (or Kruskal-Wallis test when data not normal) for univariate analysis. A multivariate ordered logistic regression model was performed to analyse the use of HBPM to diagnose and monitor blood pressure, taking gender, age, function of clinical tutor, location and type of medical practice as covariates. Results are expressed as odds ratio (OR) and its 95% confidence interval.

## Results

### Details of general practitioners

**Fig. 1:**
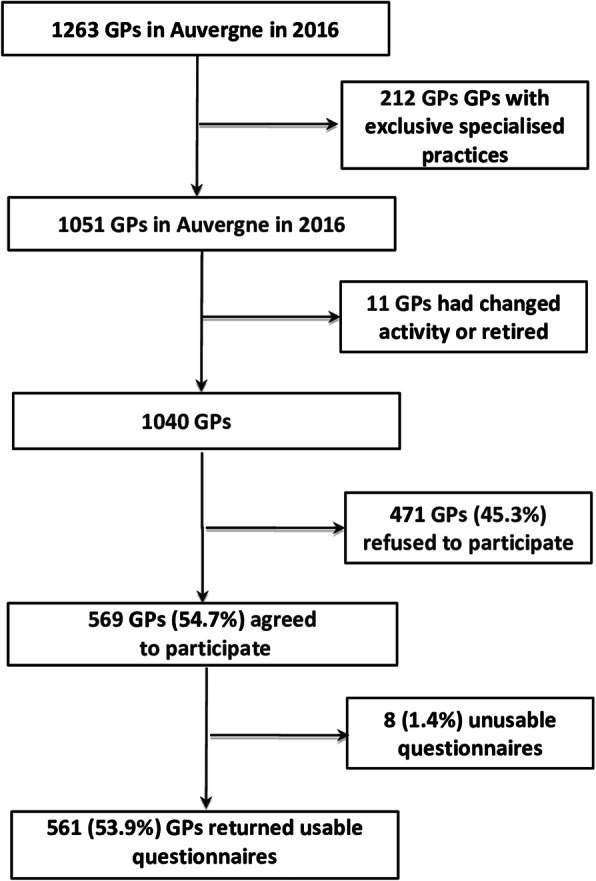
Flowchart of GPs. Legend GPs: General Practitioners

Of 1040 questionnaires sent, 569 GPs responded (54.7%) and 561 questionnaires were completed fully (Fig. 1). Characteristics of participants are described in Table 1. GPs practising in rural areas (37.3%; *p* = 0.004) and Clinical tutors (27.3%; *p* < 0.001) were better represented than in the Auvergne region as a whole and they were 2.4 years younger (50.3 vs 52.7, *p*< 0.001).

**Table 1:** Characteristics of General Practitioners

Variables	Sample *n* = 561	Overall 2016^a^ *n* = 1263	*p*-value
Women, n(%)	241 (43.0)	508 (40.2)	0.27
Age, *mean (sd)*	50.3 ± 11.5	52.7 ± 11.2	< 0.001
Department			
Allier, n(%)	141 (25.1)	312 (24.7)	0.83
Cantal, n(%)	65 (11.6)	140 (11.1)	
Haute-Loire, n(%)	79 (14.1)	199 (15.8)	
Puy-de-Dôme, n(%)	276 (49.2)	612 (48.5)	
Location			
Rural, n(%)	209 (37.3)	385 (30.5)	0.004
Urban, n(%)	352 (62.7)	878 (69.5)	
Type of practice			
Individual practice, n(%)	210 (37.4)	582 (46.1)	< 0.001
Group Practice, n(%)	296 (52.8)	681 (53.9)	
MHC, n(%)	55 (9.8)	NC	
Clinical Tutor, n(%)	153 (27.3)	199 (15.8)	< 0.001
Type of BP			
Manual BP monitor	357 (63.6)	NA	
Automated BP monitor	33 (5.9)	NA	
Both	171 (30.5)	NA	

^a^General practitioners located in the Auvergne region in 2016 (data from French National Medical Council)

b *MHC* Multidisciplinary Health Centre

### Use of home blood-pressure measurement to diagnose hypertension

Of the GPs, 530 (94.5%) declared use of HBPM to diagnose hypertension: 89 (16.8%) used it systematically, 290 (54.7%) regularly, and 151 (28.5%) occasionally. Thirty-one (5.5%) never used it. Their characteristics are presented in Table 2. In all, 501 GPs (89.3%) reported lending an HBPM device to patients (86.7% used an armband model, 8.5% a wrist model, and 4.8% both models). When lending the HBPM device, 449 GPs (89.2%) declared recommending the rule of three and 280 (55.9%) gave them a guidance sheet.

**Table 2:** Analysis of factors associated to HBPM use for diagnosis of hypertension (univariate and multivariate analysis)

	Univariate						Multivariate	
	Never	Occasionally	Regularly	Systematically	OR and 95% CI	*p*- value	OR and 95% CI	*p*- value
	*N* = 31	*N* = 151	*N* = 290	*N* = 89				
Sex F, n (%)	7 (22.6)	52 (34.4)	139 (47.9)	43 (48.3)	1.7 [1.24–2.35]	0.001	1.27 [0.90–1.79]	0.171
Age, mean ±SD	59.1±8.4	53.1±11.1	49.0±11.1	46.8±12.1	0.96 [0.94–0.97]	< 0.001	0.97 [0.95–0.98]	< 0.001
Urban City> 2500, n (%)	20 (64.5)	87 (57.6)	203 (70)	42 (47.2)	0.90 [0.65–1.25]	0.517	0.87 [0.62–1.22]	0.41
GP practice, n (%)								
Individual	21 (67.7)	71 (47)	95 (32.8)	23 (25.8)	Ref.		Ref.	
MHC^c^	0 (0)	7 (4.6)	35 (12.1)	13 (14.6)	3.68 [2.09–6.49]	< 0.001	2.09 [1.15–3.78]	0.015
Group	10 (32.3)	73 (48.3)	160 (55.2)	53 (59.6)	2.04 [1.45–2.87]	< 0.001	1.45 [1.00–2.10]	0.05
Clinical Tutor, n (%)	4 (12.9)	26 (17.2)	90 (31.0)	33 (37.1)	2.10 [1.47–3.00]	< 0.001	1.92 [1.33–2.79]	0.001

*Abbreviations*: *HBPM* Home blood pressure measurement, *GP* General practitioner, *MHC* Multidisciplinary Health Centre, *OR* Odd ratio, *RR* Relative risk, *95%CI* 95% confidence intervals

To confirm a diagnosis of hypertension, 477 GPs (85.0%) reported repeating the BP measurements in their surgery, 421 (75.0%) recommended HBPM if patients owned a device, 471 (83.9%) lent HBPM devices to patients, and 443 (78.9%) referred patients for ambulatory blood-pressure measurement with a cardiologist. Of the 530 GPs who reported the use of HBPM for diagnosis, 492 GPs (92.8%) reported its use to eliminate the ‘white-coat’ effect, 371 (70.0%) to diagnose masked hypertension, and 440 (83.0%) to adjust treatment.

To measure BP in their surgeries, 357 GPs (63.6%) reported use of a manual device, 33 (5.8%) an automated device, and 171 (30.4%) both. For 435 (86.6%) this was an armband device, for 24 (4.7%) a wrist device, for 43 (8.5%) both.

Univariate analysis reveals that female, younger, GPs practicing in groups and Multidisciplinary Healthcare Centres (MHC) and Clinical Tutors were more likely to use HBPM to diagnose hypertension (see Table 2). On multivariate analysis, younger GPs (OR = 0.97; 95% CI: 0.95–0.98), GPs practicing in group (OR = 1.45; 95% CI: 1.00–2.10) and MHC (OR = 2.09; 95% CI: 1.15–3.78), as compared to GPS with individual practice, and Clinical Tutors as compared to non-Clinical Tutors (OR = 1.92; 95% CI: 1.33–2.79) were found to use HBPM more frequently.

### Use of home blood-pressure measurement to monitor patients with hypertension

Of the respondents, 519 (92.5%) declared the use of HBPM to monitor patients: 26 (5.0%) used it systematically, 236 (45.4%) regularly, 257 (49.5%) occasionally, and 42 (8.0%) never. In all, 405 GPs (72.2%) said that they recommended patients with hypertension to buy an HBPM device.

In univariate analysis, female and younger GPS, those practicing in groups and MHC and Clinical Tutors reported more frequent use of HBPM to monitor hypertension (see Table 3). From multivariate analysis, we found that HBPM was used more frequently by female GPs, as compared to male GPs, (OR=1.47; 95% CI: 1.04–2.06), younger GPs (OR = 0.98; 95% CI: 0.97–0.99) and Clinical Tutors, as compared to non-Clinical Tutors, (OR = 1.90; 95% CI: 1.31–2.75).

**Table 3:** Analysis of factors associated to HBPM use for monitoring of hypertension (univariate and multivariate analysis)

	Univariate						Multivariate	
	Never	Occasionally	Regularly	Systematically	OR and 95% CI	*p*-value	OR and 95% CI	*p*-value
	*N* = 42	*N* = 257	*N* = 236	*N* = 26				
Sex F, n (%)	10 (23.8)	102 (39.7)	116 (49.2)	13 (50)	1.68 [1.22–2.31]	0.002	1.47 [1.04–2.06]	0.028
Age, mean±SD	53.3±9.9	51.8±11.4	48.1±11.5	50.7±12.1	0.98 [0.96–0.9]	< 0.001	0.98 [0.97–0.99]	0.027
Urban City> 2500, n (%)	26 (61.9)	162 (63)	150 (63.6)	14 (53.9)	0.97 [0.70–1.34]	0.837	0.97 [0.69–1.36]	0.856
GP practice, n (%)								
Individual	24 (57.1)	102 (39.7)	74 (31.4)	10 (38.5)	Ref		Ref	
MHC	15 (35.7)	138 (53.7)	129 (54.7)	14 (53.9)	1.49 [1.06–2.09]	0.022	1.13 [0.77–1.64]	0.536
Group	3 (7.1)	17 (6.6)	33 (14)	2 (7.7)	2.35 [1.33–4.16]	0.003	1.47 [0.80–2.70]	0.213
Clinical Tutor, n (%)	4 (9.5)	59 (23)	82 (34.8)	8 (30.8)	1.95 [1.37–2.78]	< 0.001	1.90 [1.31–2.75]	0.001

*Abbreviations*: *HBPM* Home blood pressure measurement, *GP* General practitioner, *MHC* Multidisciplinary Health Centre, *OR* Odd ratio, *RR* Relative risk, *95%CI* 95% confidence intervals

### Impact of the measures of the French National Health Insurance Body

Of the participants, 493 (87.8%) declared they had ordered the HBPM device offered on the FNHIB website. This measure enabled 309 participants (63.6%) to initiate HBPM among their patients and 76% to have an additional device.

## Discussion

Among the 561 participants who completed the survey questionnaire, 94.5% indicated that they had used HBPM to diagnose hypertension and 92.5% stated that they had used it to monitor patients with hypertension at least occasionally. Younger GPs and Clinical Tutors were more likely to use HBPM to diagnose and manage hypertension, while more GPs practising in groups and MHC reported the use of HBPM for diagnosis, and more female GPs for monitoring. To confirm hypertension, 89.3% of the GPs reported lending an HBPM device to patients. Of these, 89.2% declared that they had recommended the ‘rule of three’, according to the recommendations in France.

As in previous surveys [9, 16], somewhat surprisingly the GPs report the use of HBPM “regularly and systematically” to patients to diagnose or monitor their hypertension with electronic devices whilst also using manual devices at their own surgeries. This may be explained by the time required to obtain a correct estimate of BP in a consultation, the confidence they have in out-of-office measurements [17], and by a desire to promote self-care [18].

Of the participants, 493 (87.8%) said that they had ordered the HBPM device offered by the FNHIB. This proportion should be compared with the 1030 HBPM devices (i.e., 81.5% of GPs practising in the Auvergne region, including GPs with specialist practices) supplied by the FNHIB. This measure enabled 63.6% of the GPs to initiate HBPM use.

A previous French survey carried out in 2011 showed that 92% of GPs declared the use of HBPM occasionally, 36% to monitor and 25% to diagnose hypertension. In comparison with our survey, this reflects an increasing use of HBPM over the past 10 years [19, 20]. The French data are consistent with those obtained from other European surveys [7, 19, 21, 22]. GPs report that they use it more commonly and more in accordance with the recommendations in place in their own countries.

In our survey, 94.5% of the GPs used HPBM to diagnose hypertension and 92.5% to monitor. These results are better than those see in other studies carried out in the UK in 2016 and in Hong Kong in 2020, with respectively 22.2 and 58% to diagnose, 56.8 and 84% to monitor [7, 9]. One of the explanations for this is the likely impact of the incentives offered by FNHIB. Two web-based surveys in the UK confirmed that GPs have become more likely to use HBPM to diagnose hypertension since the introduction of new national guidance in 2011 [23, 24]. Some studies also found a wider use of HBPM to diagnose hypertension by young GPs, those working in groups or MHC, and clinical tutors [10, 12]. These are probably important factors in the promotion of HBPM.

In other countries, HBPM seems to be more likely to be used for monitoring than for diagnosis compared to our survey [7, 9]. This can be explained by differences in the healthcare systems. The participation of medical assistants and nurse prescribers in the follow-up of chronic diseases could be important for increasing the practice of HBPM to manage hypertension [25]. In France, this system is currently less well developed, with the GP carrying out the monitoring most of the time alone. The discrepancy between the number of GPs who used HBPM for diagnosis and those who used it for monitoring can also be explained by barriers related to both the patient (anxiety, purchase of equipment, difficulties in understanding the protocol, etc.) and the physician (lack of time, lack of knowledge about the evidence for the HBPM, concern about increasing patient BP-related anxiety and associated office visits or phone calls, etc.) [12, 26].

The proportions of GPs who report the use of HBPM to exclude or confirm so-called ‘white-coat’ effects and masked hypertension are similar to those seen in our own survey. Compared to older surveys [19, 27], the practice of blood pressure self-measurement is now more common among GPs.

Studies have also shown that the majority of GPs recommended that their patients record their HBPM, sometimes by lending a device to the patient, as in our survey [21, 27–29]. In parallel, surveys have also revealed that an increasing number of patients in primary care or specialist centres use self-monitoring of blood pressure [22–24, 29]. The conditions of use are not always in accordance with the recommendations, however. In our survey, 89.5% of participants declared teaching the ‘rule of three’ to their patients. This is a significant increase compared to the survey of Boivin et al. (2009), where just 17% recommended this rule to patients [19].

Our cross-sectional survey is justified for evaluating the practice of HBPM as recommended by GPs, 3 years after the introduction of the FNHIB incentives in the pilot region. All GPs in the Auvergne region were contacted in order to be as precise as possible in meeting the main objective and to ensure significant results. This made it possible to avoid the use of stratified randomisation to obtain a representative sample of the population of GPs in the Auvergne area. The questionnaire was intentionally short to encourage a good response rate.

Our study has several limitations, the main one being is the modest response rate; only 54.7% of GPs responded to the questionnaire. The practice and awareness of non-responders are not known and it is likely that only the most interested and those who used HBPM most frequently participated. Our response rate is in accordance with the average response rate for mail surveys of GPs [30], including those related to hypertension [19, 31]. Recent web surveys have different response rates, from 20 to 89% [9, 21, 27, 28, 32], with easier follow-up opportunities, but response rates are not directly comparable to postal surveys, because it is impossible to know how many people saw the hyperlink but did not click on it.

The responding sample was similar to the total population with regard to sex and distribution across counties, but the participants were around 2.4 years younger and Clinical Tutors and rural GPs were over-represented. These 2 categories reported a greater utilization of HBPM, so this is probably an overestimate of the number of declared users.

An additional bias is seen in the fact that ours is a descriptive study using a self-completed questionnaire. The data collected were declarative and do not necessarily correspond to the reality of the practice concerned. Because the questionnaire was anonymous, it was not possible to know which GPs had responded and not possible to send reminders to those who had not.

The practice of HBPM by French GPs is becoming widespread. The provision of the FNHIB is an incentive but it is insufficient on its own to increase the use of HBPM. Medical practices must equip themselves with several devices and organise their daily allocation. Because it remains a useful tool for self-care, HBPM requires GP’s involvement in promoting the method, explaining the rules for correct use to patients and interpreting the results [7]. This self-measurement learning could be delegated to nurses and/or pharmacist as part of therapeutic education programs [33, 34].

## Conclusions

Three years after introducing the incentives of French National Health Insurance Body to the Auvergne region, most respondents indicated that the use of HBPM to diagnose and monitor hypertension according to the recommendations is high. This measure has enabled a significant number of GPs to initiate HBPM among their patients. Younger GPs and Clinical Tutors were more likely to use HBPM for both diagnosis and monitoring of hypertension. We therefore expect its more widespread use in future. Further studies involving GPs and patients are necessary to confirm the increasing use of HBPM in France as a whole.

## Supplementary Information

**Additional file 1.**

## Data Availability

The datasets used and/or analysed during the current study are available from the corresponding author on reasonable request.

## Abbreviations

ABPM: Ambulatory Blood Pressure Measurement
BP: Blood Pressure
CFLHTA: French League Against Hypertension
FNHIB: French National Health Insurance Body
GPs: General Practitioners
HBPM: Home Blood Pressure Measurement
MHC: Multidisciplinary Healthcare Centres
