# Supplementary material for: Home blood-pressure measurement for the diagnosis and monitoring of arterial hypertension by French general practitioners: a cross-sectional survey in the Auvergne region
Source: BMC Fam Pract. 2021 Jan 4;22:7. doi: 10.1186/s12875-020-01358-9 (PMC7780393; doi:10.1186/s12875-020-01358-9)
Supplement: Supplementary file 1 — Additional file 1. [file 12875_2020_1358_MOESM1_ESM.docx]

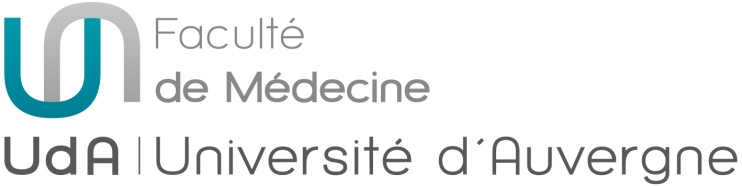

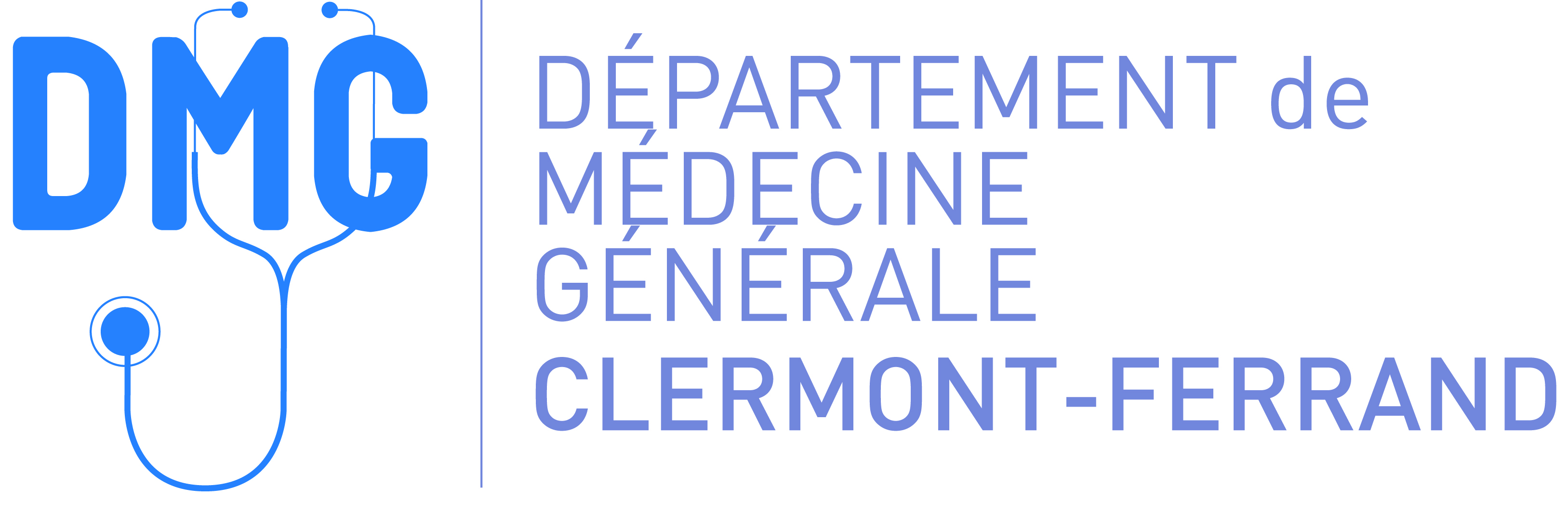


**PANORAMA
Place de l’AutOmesuRe de la tension Artérielle en Médecine générale en Auvergne**

**My profile of General Practitioner**

1/ I am : Woman / Man

2/ My age :

3/ I practice in the department of :

Allier / Cantal / Haute-Loire / Puy-de-Dôme

4/ I practice in a community of :

- Less than 2500 inhabitants

- More than 2500 inhabitants

5/ I practice :

- Individual practice

- Group practice

- Multidisciplinary health centre

6/ I am a GP teacher : Yes / No

**Blood pressure measurement in my practice**

7/ For blood pressure measurement in the office, I use :

A manual blood pressure monitor / An automated blood pressure monitor / Both

8/ I have an HBPM device to lend to my patients? Yes / No

if so,

- Number of devices

- Type of device : Arm / Wrist / both

- I recommend the “rule of 3” (3 measurements in the morning and in 3 measurements the evening X 3 days) : Yes / No

- I give the patient a tip sheet : Yes / No

9/ I have an ambulatory blood pressure monitor (ABPM) for 24-hour BP monitoring Yes / No

10/ I confirm the diagnosis of hypertension (several responses possible):

- With another control at the office : Yes / No

- By HBPM at the patient's home, if the patient possesses an HBPM device : Yes / No

- I lend the patient an HBPM device : Yes / No

- I sometimes recommend 24-hour ABPM : Yes / No

11/ I use HBPM for the diagnosis of hypertension : Never / Occasionally / Regularly / Systematically

12/ For the monitoring of hypertension ( several responses possible)

- I adapt the treatment according to the BP measurement at the office : Yes No

- I adapt the treatment according to an HBPM : Yes / No

if so,

- I recommend that patients purchase an HBPM device : Yes / No

- I lend the patient an HBPM device : Yes / No

13/ I use the HBPM for the follow-up of patients

Never / Occasionally / Regularly / Systematically

14/ I ordered the HBPM device offered free of charge by the French National Health Insurance Body

Yes /No

15/ The availability of this device allowed me to

- To initiate HBPM with my patients : Yes / No

- To have an additional device for my regular HBPM practice : Yes / No

**My opinion on the HBPM**

16/ Has no practical relevance. Yes / No /IDK

17/ May cause anxiety for patients. Yes /No /IDK

18/ Is too time-consuming to explain. Yes /No /IDK

19/ Is too difficult for some patients. Yes /No /IDK

20/ Represents too much money investment. Yes /No /IDK

21/ Unfortunately not listed by the National Health Insurance Body. Yes /No /IDK

22/ Avoids the white coat effect. Yes /No /IDK

23/ Allows to diagnose masked hypertension. Yes /No /IDK

24/ Allows the patient to take care of himself. Yes /No /IDK

25/ Promotes drug compliance. Yes /No /IDK

26/ Allows to confirm the resistant character of the hypertension. Yes /No /IDK

27/ Is essential in adjusting the treatment of some patients. Yes /No /IDK

28/ Material problems (lost patients, loss or deterioration of device) have delayed me in his implementation. Yes /No /IDK

29/ In total, I would say that HBPM in my daily practice is :

Unnecessary / Somewhat useful / Very useful / Indispensable
